# Shedding light on a Group IV (ECF11) alternative σ factor

**DOI:** 10.1111/mmi.14280

**Published:** 2019-05-31

**Authors:** Timothy J. Donohue

**Affiliations:** ^1^ Bacteriology Department, Great Lakes Bioenergy Research Center Wisconsin Energy Institute, University of Wisconsin‐Madison Madison WI 53726 USA

## Abstract

This year marks the 50^th^ anniversary of the discovery of σ^70^ as a protein factor that was needed for bacterial RNA polymerase to accurately transcribe a promoter *in vitro*. It was 25 years later that the Group IV alternative σs were described as a distinct family of proteins related to σ^70^. In the intervening time, there has been an ever‐growing list of Group IV σs, numbers of genes they transcribe, insight into the diverse suite of processes they control, and appreciation for their impact on bacterial lifestyles. This work summarizes knowledge of the *Rhodobacter sphaeroides* σ^E^‐ChrR pair, a member of the ECF11 subfamily of Group IV alternative σs, in protecting cells from the reactive oxygen species, singlet oxygen. It describes lessons learned from analyzing ChrR, a zinc‐dependent anti‐σ factor, that are generally applicable to Group IV σs and relevant to the response to single oxygen. This MicroReview also illustrates insights into stress responses in this and other bacteria that have been acquired by analyzing or modeling the activity of the σ^E^‐ChrR across the bacterial phylogeny.

## Graphical abstract

Activation of a cascade of alternative σ factors by the reactive oxygen species ^1^O_2_. The master regulator of this response, the Group IV ECF11 protein *Rb. sphaeroides* σ^E^, directly activates transcription of ~genes, one (*rpoH_II_*) encodes an alternative σ factor in the heat shock family. RpoH_II_ directly activates ~145 genes; some 45 of which are also transcribed by RpoH_I_, the master regulator of the* Rb. sphaeroides* heat shock response.

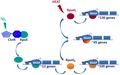

## Introduction

The study of Group IV or extracytoplasmic function (ECF) σ factor function has provided many new insights into the cell biology, stress responses and signaling pathways across the bacterial phylogeny (Staron *et al.*, 2009; Feklístov *et al.*, 2014), and provided strategies to allow for targeted control of gene expression in native and heterologous hosts (Rhodius *et al.*, 2013; Pinto *et al.*, 2019). This contribution will review what is known about the ECF11 sub‐family of Group IV σs (Staron *et al.*, 2009). It will focus on the founding member of the ECF11 sub‐family, the *Rhodobacter sphaeroides* σ^E^ protein, its cognate cytoplasmic anti‐σ ChrR (Newman *et al.*, 1999; 2001), and their role in a stress response to singlet oxygen, a reactive oxygen species (ROS) encountered by a variety of cells (Anthony *et al.*, 2005; Ziegelhoffer and Donohue, 2009). It will summarize lessons learned about Group IV σ factor function by studying this system, and highlight unanswered questions about this response.

### Singlet oxygen (^1^O_2_) is a ROS

Prior to the introduction of molecular oxygen (O_2_), organisms had a limited metabolic and regulatory repertoire. However, when photosynthetic cells acquired the ability to produce O_2_, they altered the Earth's atmosphere and influenced the forms of life that inhabited the planet (Raymond *et al.*, 2003; Kerr, 2005). In particular, the accumulation of atmospheric O_2_ allowed evolution of pathways like aerobic respiration that couple the four‐electron reduction of O_2_ to formation of a proton gradient (Kerr, 2005). One advantage to aerobic respiration is the large amount of energy that is conserved as O_2_ is reduced to water (Gennis, 1986; Brzezinski and Gennis, 2008; Borisov *et al.*, 2011; Bueno *et al.*, 2012; Soo *et al.*, 2019). However, there are other, potentially deleterious, consequences to life in the presence of O_2_. One trade off to accumulation of atmospheric O_2_, or its use as a terminal electron acceptor, is formation of different ROS (Rosner and Storz, 1997; Schulz *et al.*, 2000; Mittler *et al.*, 2004; Frick *et al.*, 2015; Taverne *et al.*, 2018).

**Figure 1:**
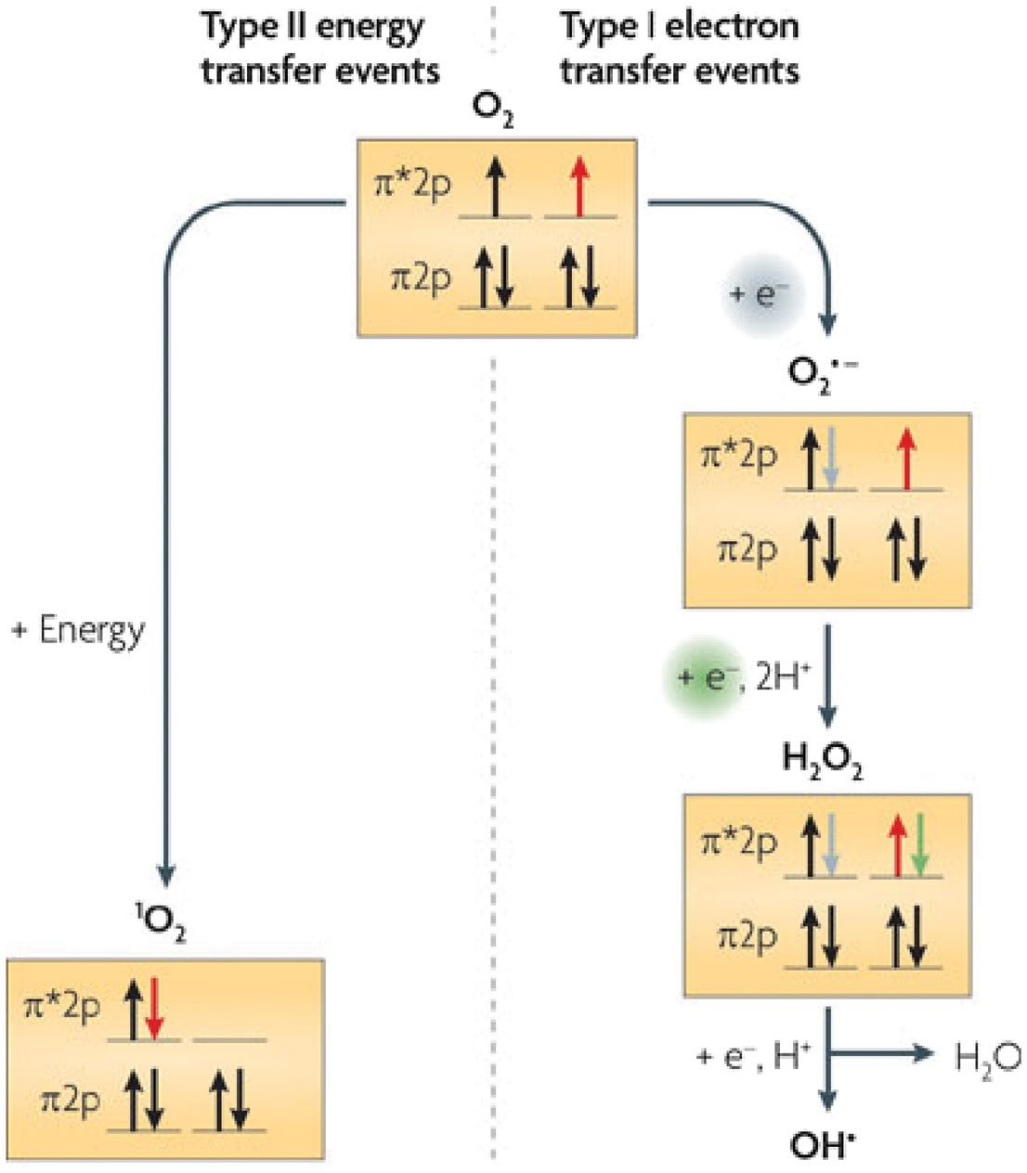
Formation and biological consequences of ROS generation. The right panel shows production of the ROS superoxide (O_2_^•−^), hydrogen peroxide (H_2_O_2_) or hydroxyl radicals (OH^•^) by one‐electron transfer reactions. The left panel shows formation of singlet oxygen (^1^O_2_) by Type II energy transfer, typically from an excited, triplet state donor, to O_2_. The diagrams show the spin of electrons in shells of the outer *p* orbital of each compound. Note that ^1^O_2_ is formed by movement of an electron between outer *p* orbital shells (red arrow). Figure modified from (Ziegelhoffer and Donohue, 2009).

When one electron is sequentially transferred to O_2_ (Fig. 1), Type I ROS (superoxide, hydrogen peroxide, or hydroxyl radicals) are formed (Rosner and Storz, 1997). Each Type I ROS can damage biomolecules, kill cells or trigger the onset of debilitating diseases (Schulz *et al.*, 2000; Taverne *et al.*, 2018). Consequently, considerable effort has been invested into determining the stress response(s) to these ROS (Rosner and Storz, 1997; Schulz *et al.*, 2000; Zheng and Storz, 2000; Kiley and Storz, 2004; Taverne *et al.*, 2018).

In contrast, less is known about how cells respond to the Type II ROS ^1^O_2_ (Ragàs *et al.*, 2013; Dogra *et al.*, 2018). ^1^O_2_ is formed when energy transfer from an excited, triplet state donor, to O_2_ alters the distribution of electrons in its outer orbital (Fig. 1). Enzymes which detoxify superoxide or H_2_O_2_ are ineffective against ^1^O_2_ due to differences in outer orbital electron organization between these Type 1 and Type II ROS (Ziegelhoffer and Donohue, 2009). Indeed, there are no known enzyme‐catalyzed systems for ^1^O_2_ detoxification (Davies, 2004).

The outer orbital electron organization of ^1^O_2_ makes it a strong oxidant (~900mV energy difference between ^1^O_2_ and O_2_). ^1^O_2_ is known or predicted to peroxidize and eventually cleave unsaturated bonds in olefins, oxidize amino acid side chains or nucleic acid bases and cleave peptide or phosphodiester bonds (Nymann and Hynninen, 2004; Godley *et al.*, 2005). Thus, it is not surprising that ^1^O_2_ can also inhibit growth or kill cells (Anthony *et al.*, 2005; Ziegelhoffer and Donohue, 2009; Lemke *et al.*, 2014).

### Biological formation of ^1^O_2_

Major cellular sources of ^1^O_2_ include the enzymes NADH oxidase, myloperoxidase or chloroperoxidase (Kochevar, 2004; Davies, 2004; Godley *et al.*, 2005). Light energy capture by photosynthetic pigments is another significant source of ^1^O_2_ (Fig. 1). In the light reactions of photosynthesis, photons excite chlorophyll pigments to a high‐energy state (Cogdell, 2000; Frank and Brudvig, 2004; Kochevar, 2004; Triantaphylides and Havaux, 2009). Normally, these excited (triplet state) pigments transfer energy to a reaction center (in bacteria) or photosystem (in cyanobacteria, algae and plants) resulting in light‐driven oxidation of this membrane enzyme. However, at a significant frequency, energy transfer from light‐excited photopigments to O_2_ generates ^1^O_2_ (Cogdell, 2000; Frank and Brudvig, 2004; Kochevar, 2004; Uchoa *et al.*, 2008; Triantaphylides and Havaux, 2009).

^1^O_2_ has a high reactivity, so it is predicted to have a short cellular half‐life (~100 ns), not travel far its site of synthesis, and produce localized damage (Kochevar, 2004). In phototrophs, ^1^O_2_ formation initiates a process called photo‐oxidative stress (Triantaphylides and Havaux, 2009; Ziegelhoffer and Donohue, 2009) that can inactivate integral photosynthetic membrane enzymes (Cogdell, 2000; Fryer *et al.*, 2002; Frank and Brudvig, 2004; Kochevar, 2004; Szabó *et al.*, 2005), peroxidize or cleave nearby olefins (carotenoids or unsaturated fatty acids), destroy bilayer integrity and function (Girotti and Kriska, 2004; Ramel *et al.*, 2012; Lemke *et al.*, 2014), signal changes in nuclear gene expression from the organelle (chloroplasts) where it is generated in eukaryotic phototrophs or trigger apoptosis (Danon *et al.*, 2005; Foyer and Noctor, 2005).

### ^1^O_2_ promotes a bacterial transcriptional response

**Figure 2:**
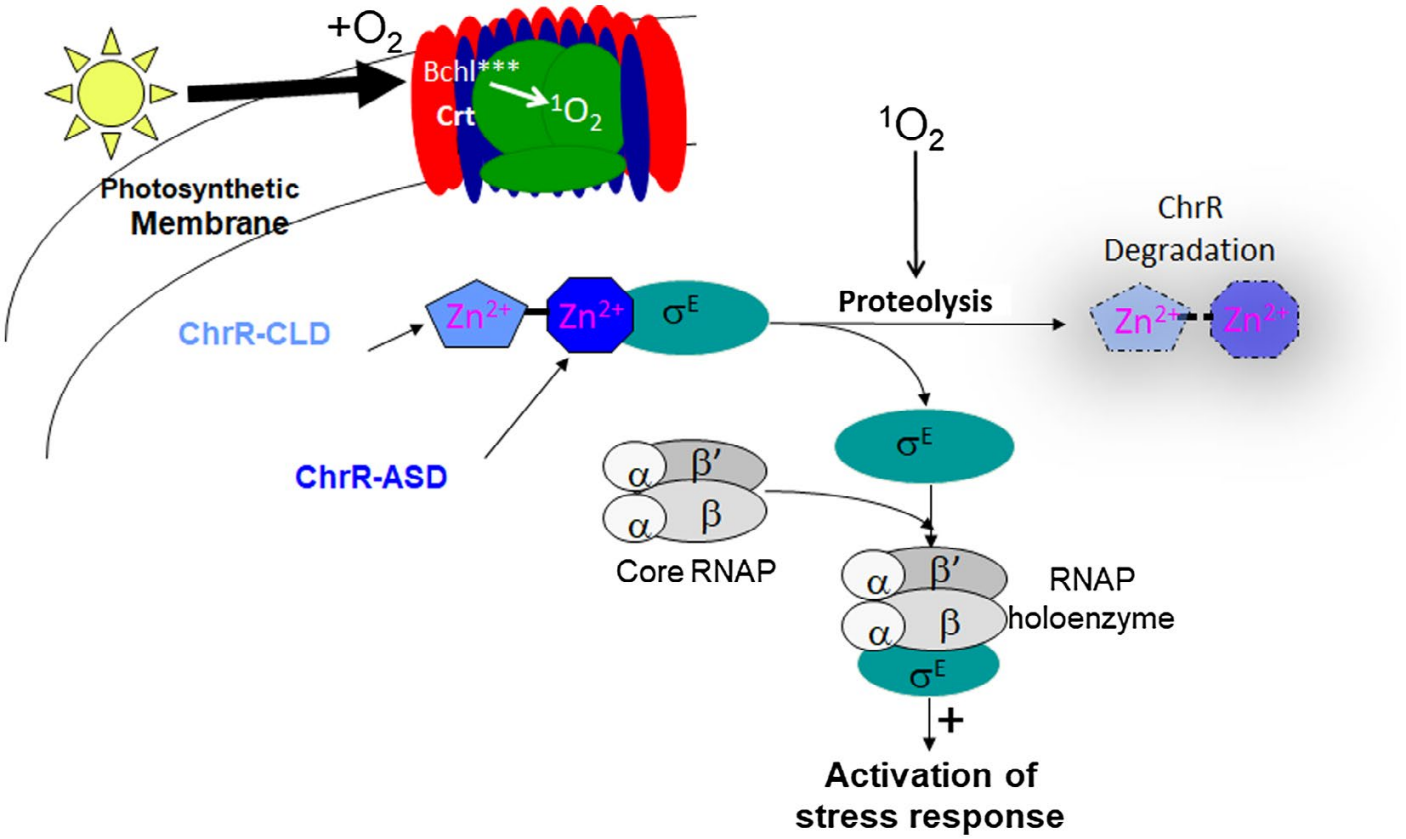
Model for activation of the *Rb. sphaeroides* σ^E^‐dependent ^1^O_2_ stress response. *Top* Depicts formation of ^1^O_2_ during light‐driven energy transfer from excited pigments (Bchl^***^) of the photosynthetic membrane to O_2_. *Middle* Depicts the ability of ^1^O_2_ to somehow (signal unknown) promote ChrR degradation, releasing the Group IV sigma factor σ^E^, so it binds RNA polymerase (RNAP) and directly activates transcription of genes in the resulting stress response. ChrR is color‐coded to denote interactions between its N‐terminal ASD domain with σ^E^ and the C‐terminal ChrR‐CLD (see text).

We uncovered a role for an ECF11 Group IV σ factor in a ^1^O_2_ stress response by studying the photosynthetic bacterium *Rb. sphaeroides* (Anthony *et al.*, 2005; Dufour *et al.*, 2008; Ziegelhoffer and Donohue, 2009; Nam *et al.*, 2013). In the laboratory, photosynthetic growth of* Rb. sphaeroides* is often achieved by incubating cells anaerobically in the light (Tavano and Donohue, 2006; Donohue and Kiley, 2011), so ^1^O_2_ is not formed under these conditions. However, we discovered that* Rb. sphaeroides* mounts a transcriptional response to ^1^O_2_ either when pigmented cells are exposed to light and O_2_ or when non‐pigmented cells are exposed to the photosensitizer methylene blue, light and O_2_ (Anthony *et al.*, 2005), two conditions that are well known to produce this ROS (Fig. 2). The master regulator of this transcriptional response to ^1^O_2_ in *Rb. sphaeroides* is the Group IV σ factor, σ^E^ (Anthony *et al.*, 2005; Campbell *et al.*, 2007; Greenwell *et al.*, 2011).

In many cells, the ability of carotenoids to quench ^1^O_2_ is generally accepted to be a major route of detoxification of this ROS (Cogdell, 2000; Frank and Brudvig, 2004; Kochevar, 2004). However, quenching by carotenoids must not provide complete protection against ^1^O_2_ since this ROS can inactivate proteins, and oxidize membrane fatty acids and other olefins (Rinalducci *et al.*, 2004; Kochevar, 2004; Nishiyama *et al.*, 2004; Estevam *et al.*, 2004; Ramel *et al.*, 2012; Lemke *et al.*, 2014). In addition, ^1^O_2_ formation kills *Rb. sphaeroides* Δσ^E^ cells, demonstrating the essential role of the σ^E^‐dependent transcriptional response to this ROS (Anthony *et al.*, 2005).

The *Rb. sphaeroides* σ^E^‐dependent pathway is not activated by superoxide, H_2_O_2_ or hydroxyl radicals (Anthony *et al.*, 2005; Greenwell *et al.*, 2011). However, we and others found that the organohydroperoxide *tert*‐butylhydroperoxide (*t*‐BOOH) increases *Rb. sphaeroides* σ^E^ activity (Lourenco and Gomes, 2009; Greenwell *et al.*, 2011; Nam *et al.*, 2013). Based on studies with model compounds, ^1^O_2_ oxidization of biomolecules could form organohydroperoxides in the membrane (Stief, 2003; Davies, 2004; Kochevar, 2004; Watabe *et al.*, 2007; Triantaphylides and Havaux, 2009). Thus, activation of the *Rb. sphaeroides* σ^E^ pathway by either ^1^O_2_ or *t*‐BOOH could reflect signal correlation, an evolutionary adaptation that allows cells to mount a response to two membrane‐localized signals (^1^O_2_ and organohydroperoxides) that are often found together in nature (Dufour *et al.*, 2010; 2012; Dufour and Donohue, 2012). Several candidate enzymes that could detoxify *t*‐BOOH or its oxidation products are part of the *Rb. sphaeroides* σ^E^‐dependent stress response (Dufour *et al.*, 2012; Dufour and Donohue, 2012).

### ChrR is a negative regulator of *Rb. sphaeroides* σ^E^ activity

Group IV σs typically bind to a cognate an anti‐σ factor that is co‐transcribed with the σ factor structural gene (Staron *et al.*, 2009). *Rb. sphaeroides* σ^E^ follows this paradigm since ChrR, its cognate anti‐σ, which forms a complex with σ^E^ that prevents it transcribing target genes (Newman, 2001; Newman *et al.*, 2001; Anthony *et al.*, 2004; Campbell *et al.*, 2007), is encoded by the *rpoEchrR* operon (Newman *et al.*, 1999). However, unlike many other Group IV anti‐σ factors, which are integral membrane proteins (Staron *et al.*, 2009), *Rb. sphaeroides* ChrR is a cytoplasmic protein (Newman *et al.*, 2001; Anthony *et al.*, 2003; 2004; Campbell *et al.*, 2007). Indeed, *Rb. sphaeroides* ChrR was the founding member of the ECF11 family of Group IV anti‐σs (Staron *et al.*, 2009).

### How ChrR blocks σ^E^ activity

**Figure 3:**
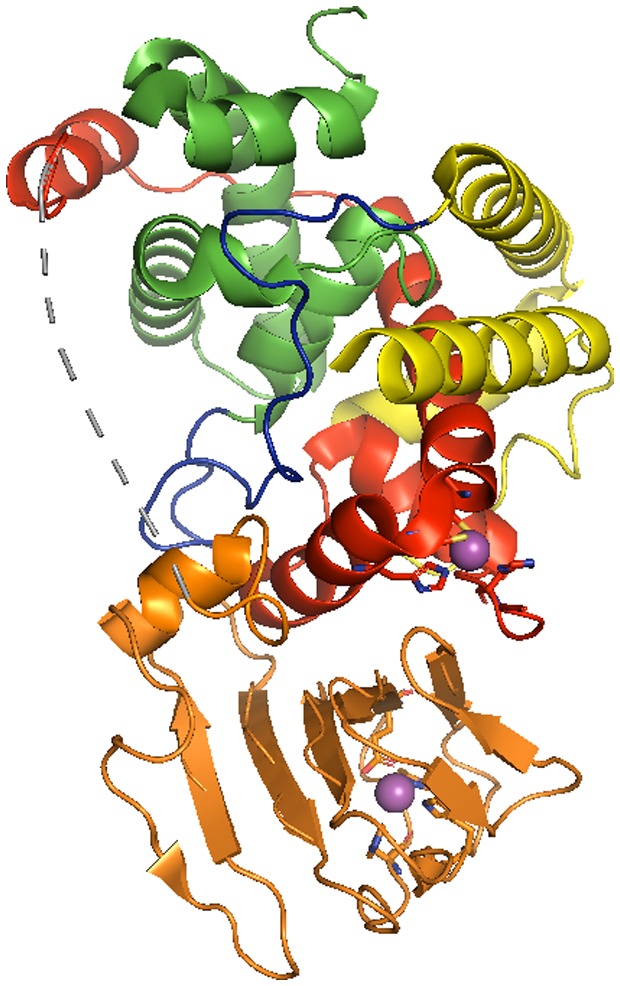
Structure of the *Rb. sphaeroides* σ^E^‐ChrR complex. The *Rb. sphaeroides* σ^E^ protein is colored to reflect the major functional domains of sigma factors (green‐region 2; blue‐region 2‐4 linker; yellow‐region 4). ChrR is colored to indicate its two major structural domains (red‐the 4 helical bundle that contains the N‐terminal anti‐sigma domain, ASD; orange‐the C‐terminal cupin‐like domain, CLD). Note the extensive interactions of the ChrR‐ASD with σ^E^ regions 2 and 4. The 2 zinc atoms associated with ChrR are shown in magenta, along with the side chains of the amino acids that ligate these metals (^6^His, ^31^His, ^35^Cys & ^38^Cys in the ChrR‐ASD and ^141^His, ^143^His, ^147^Glu and ^177^His in the ChrR‐CLD respectively).

Key insights into the ECF11 family came from solving the three‐dimensional structure of the *Rb. Sphaeroides* σ^E^‐ChrR complex (in collaboration with the Darst lab) (Campbell *et al.*, 2007). In the σ^E^‐ChrR complex, the *Rb. sphaeroides* σ^E^ fold is similar to that of other σs, including *Escherichia coli* σ^70^ and σ^E^ (Campbell *et al.*, 2002; 2003), consisting of two α‐helical domains (σ regions 2 and 4) connected by a short domain 2‐4 linker (Fig. 3).

In this structure, ChrR contains two major structural elements that were connected by a flexible linker. The ChrR N‐terminal domain makes extensive contacts with those σ^E^ regions predicted to bind RNA polymerase and promoter DNA (Fig. 3), so this part of ChrR was called the anti‐sigma domain (ChrR‐ASD) to denote how it could block σ factor function.

### Zinc binding to the ChrR‐ASD is needed to inhibit σ^E^ activity

*Rb. sphaeroides* ChrR and *Streptomyces coelicolor* RsrA were founding members of the ZAS anti‐σ proteins, a family of zinc‐dependent anti‐sigma factors that each bind a zinc metal in their N‐terminal ASDs (Paget *et al.*, 2001a; Paget and Buttner, 2003; Bae *et al.*, 2004; Zdanowski *et al.*, 2006). It was known that zinc binding was required for ChrR to inhibit σ^E^ activity (Newman *et al.*, 2001). In the σ^E^:ChrR complex, zinc is tetrahedrally coordinated to amino acid side chains in the ChrR‐ASD (Fig. 3) that were predicted to be involved in zinc binding based on studying mutant ChrR proteins containing single alanine substitutions at these positions (Newman *et al.*, 2001).

### Conservation of structure and function among different Group IV anti‐σs

**Figure 4:**
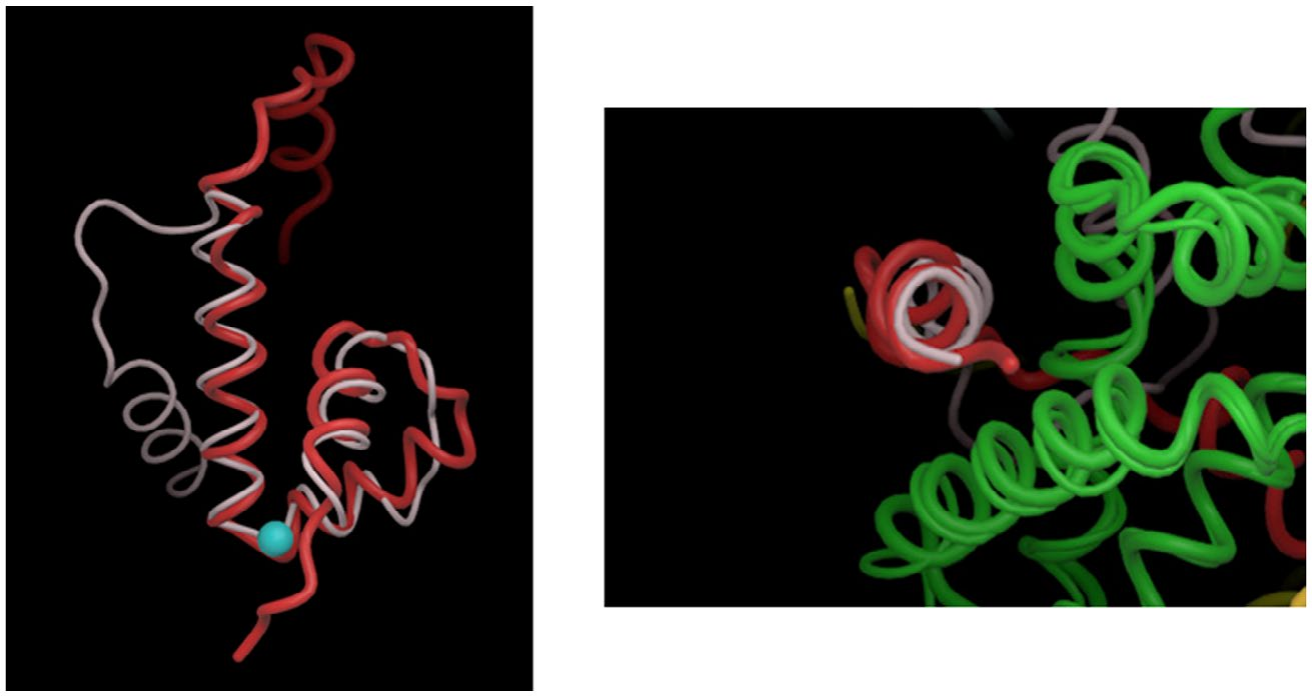
Structural similarity between the *Rb. sphaeroides* ChrR‐ & *E. coli* RseA‐ASD. The left panel shows the structural similarity between helices I‐III and the displacement of helix IV of the ASD of *Rb. sphaeroides* ChrR (red) and *E. coli* RseA (white). The blue sphere is the Zn^2+^ atom in the ChrR‐ASD. The right panel shows that, despite this displacement of helix IV in the ASD of ChrR (red) and RseA (white), it interacts with a structurally conserved part of region 2 in the cognate Group IV sigma factors (region 2 of the *Rb. sphaeroides* and *E. coli* σ^E^ proteins are both shown in green). Figures modified from (Campbell *et al.*, 2007).

The structure of the σ^E^‐ChrR complex contributed to developing an early model for how many anti‐sigma factors could inhibit function of Group IV σs (Campbell *et al.*, 2002). This model was based on the unexpected finding that the ChrR‐ASD and the N‐terminal domain of the *E. coli* anti‐sigma factor RseA (RseA‐ASD) both contain similar α‐helical bundles despite the lack of significant primary amino acid sequence similarity between these proteins (Campbell *et al.*, 2007). Each ASD contains one structurally conserved helix, helix IV, which interacts with region 2.1 of its cognate Group IV σ in their respective complexes (Fig. 4, right). Based on this, we proposed that many other ECF anti‐sigma factors will use a region structurally related to ASD helix IV to bind region 2.1 of their cognate ECF σs and block RNA polymerase binding (Campbell *et al.*, 2007).

We also made the unexpected finding that the region of structural similarity between the ChrR‐ and RseA‐ASDs includes the ChrR zinc binding site (Fig. 4, left). The ability of both anti‐σs to adopt a similar fold shows that either zinc–protein interactions (in ChrR) or protein–protein interactions (in RseA) can stabilize the ASD helical bundle (Campbell *et al.*, 2007).

The other structurally conserved helices in the ASD of each anti‐sigma factor (helices I‐III) interact with different regions of their cognate sigma factors, providing a way for each anti‐σ factor to recognize specific partner proteins (Campbell *et al.*, 2007). When these observations were combined with comparative genomics, it predicted that the ASDs of ChrR homologs, as well as many other Group IV anti‐σ factors that have lower degrees of amino acid identity and thus fall into other ECF subfamilies (Staron *et al.*, 2009), could adopt a similar fold when bound to their cognate σ factor (Campbell *et al.*, 2007). Subsequent structural analysis of additional complexes has revealed that, while the ASD conformation has been highly conserved among anti‐sigma factors, the mechanism of inhibition of σ factor activity is unique for each cognate pair examined (Sineva *et al.*, 2017).

### The C‐terminal domain of ChrR is needed to release σ^E^ in the presence of ^1^O_2_

The ChrR C‐terminal domain binds a 2^nd^ zinc atom within a structural element that adopts an overall fold similar to proteins in the cupin superfamily (Khuri *et al.*, 2001), so we called this the ChrR cupin‐like domain (ChrR‐CLD, Fig. 3). In structurally characterized proteins that contain a CLD, it can have enzyme activity (isomerases) or bind a ligand (Khuri *et al.*, 2001). The overall CLD fold and the residues known to bind zinc in the σ^E^‐ChrR structure are predicted to exist in many other ChrR homologs (Campbell *et al.*, 2007) and a variety of ZAS that are members of other ECF sub‐families (Rajasekar *et al.*, 2016).

The ChrR‐CLD had little contact with σ^E^, leading us to propose that this region was unnecessary for formation of the σ^E^‐ChrR complex. As predicted, a truncated ChrR protein lacking the CLD (ChrR85) inhibited σ^E^ activity, but cells containing ChrR85 did not mount a transcriptional response to ^1^O_2_ (Campbell *et al.*, 2007), predicting that the ChrR‐CLD is needed to activate the response. When we analyzed function of ChrR variants containing amino acid substitutions in the CLD zinc ligands, we identified side chains that are (^147^Glu and ^177^His) and are not (^187^Cys and ^189^Cys) needed for ^1^O_2_ or the organoperoxide like t‐BOOH (see below) to increase σ^E^ activity (Greenwell *et al.*, 2011).

### ^1^O_2_ stimulates ChrR turnover

Some Group IV anti‐σ factors, including others that bind zinc, are reversibly modified by an inducing signal (Paget and Buttner, 2003; Antelmann and Helmann, 2011). However, there is no known mechanism for reversible protein modification by ^1^O_2_ (Davies, 2004). Instead, we found that ^1^O_2_ promotes ChrR proteolysis (Nam *et al.*, 2013), releasing σ^E^ so it can bind RNA polymerase (Anthony *et al.*, 2004) and directly activate transcription (Ziegelhoffer and Donohue, 2009; Dufour *et al.*, 2010; 2012).

There is precedent for regulated proteolysis of a Group IV anti‐σ factor during a stress response, since cleavage of *E. coli* RseA is initiated by a protease cascade (including DegS and YaeL) that responds to envelope stress (Alba *et al.*, 2002). After DegS and YaeL cleavage of RseA in its membrane spanning region, housekeeping proteases complete degradation of this anti‐σ, releasing the *E. coli* σ^E^ so it can activate transcription (Chaba *et al.*, 2007). Homologs of extra‐cytoplasmic proteases that cleave RseA have been reported to promote ChrR turnover *in vivo* (Nuss *et al.*, 2013). However, direct proteolytic cleavage of ChrR has yet to be reported and it is not clear how membrane‐ or periplasmic‐localized proteases promote direct or indirect degradation of this cytoplasmic anti‐σ. Indeed, it is possible that ^1^O_2_ initiates a conformational change in ChrR that can makes it protease‐susceptible since this ROS can remove zinc from a synthetic peptide that contains the metal ligands and mimics the fold found in the ChrR‐ASD (Chabert *et al.*, 2019). There is precedent for zinc release playing such a regulatory role in the bacterial chaperone Hsp33 and the ZAS anti‐σ factor, RsrA, that each respond to oxidative stress signals (Jakob *et al.*, 2000; Kim *et al.*, 2001; Raman *et al.*, 2001; Paget *et al.*, 2001a; 2001b; Paget and Buttner, 2003; Zdanowski *et al.*, 2006; Rajasekar *et al.*, 2016), so the precise role(s) of the ChrR‐ASD zinc in the response to ^1^O_2_ is unknown.

Other proteins have been shown to be needed for ChrR turnover in the presence of ^1^O_2_ (Nam *et al.*, 2013; Nuss *et al.*, 2013). However, these proteins lack significant amino acid sequence similarity to proteases but some catalyze synthesis of an unusual furan‐containing fatty acid (Lemke *et al.*, 2014). ^1^O_2_ formation promotes destruction of furan‐containing fatty acids, so it has been proposed that peroxidation of membrane bound olefins can act as a second messenger to stimulate the activity of one or more proteases that initiates degradation of ChrR in response to this ROS (Lemke *et al.*, 2014).

### Organohydroperoxides and ^1^O_2_ promote ChrR turnover by different mechanisms

While the presence of either ^1^O_2_ or an organoperoxide like t‐BOOH increases σ^E^ activity (Glaeser *et al.*, 2011; Nam *et al.*, 2013), they appear to inactivate ChrR by different mechanisms. By comparing the ability of either ^1^O_2_ or *t*‐BOOH to increase σ^E^ activity in cells containing wild‐type ChrR, a truncated ChrR85 protein, variant ChrR proteins with single amino acid changes (Greenwell *et al.*, 2011), and host mutants with defects in furan fatty acid synthesis (Nam *et al.*, 2013), one can genetically separate the effects of ^1^O_2_ and t‐BOOH on σ^E^ activation. To explain this observation, it has been proposed that ChrR inactivation in the presence of ^1^O_2_ (requires a ChrR‐CLD) or *t*‐BOOH (occurs in cells lacking an intact ChrR‐CLD) do not occur by identical mechanisms (Greenwell *et al.*, 2011; Nam *et al.*, 2013).

### The biological response to ^1^O_2_

**Figure 5:**
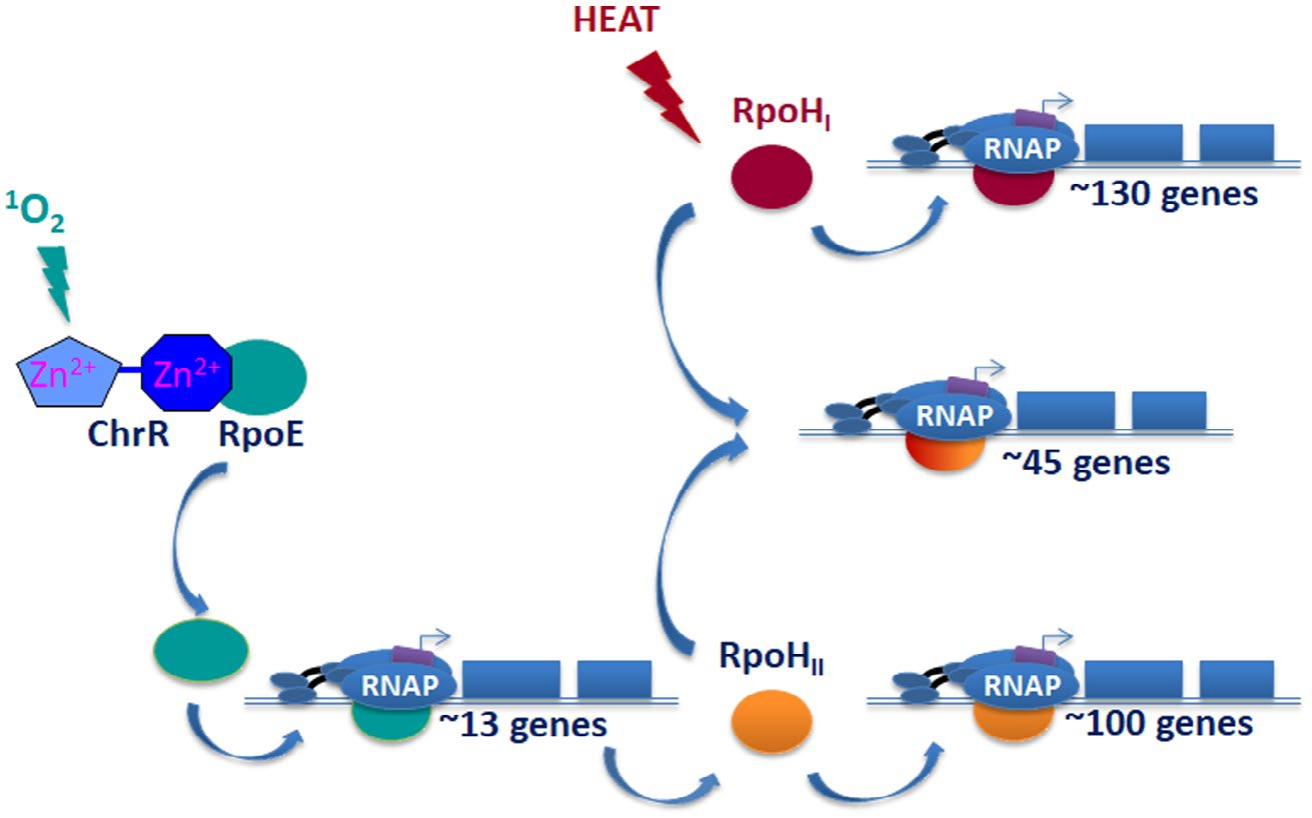
^1^O_2_ activates a transcriptional cascade. Shown is the transcriptional cascade that is activated by the presence of ^1^O_2_ in *Rb. sphaeroides*. The master regulator, σ^E^, directly activates transcription of 13 genes, one of which (*rpoH_II_*) encodes one of two *Rb. sphaeroides* alternative σ factors in the heat shock family. RpoH_II_ directly activates ~145 genes; some 45 of which are also transcribed by RpoH_I_, the master regulator of the* Rb. sphaeroides* heat shock response. Data summarized from (Dufour *et al.*, 2012).

Identifying the members of a transcriptional regulon is often instrumental to understanding functions needed during a stress response (Guisbert *et al.*, 2008). By combining computational (phylogenetic clustering), *in vitro* (transcription assays) and *in vivo* (gene fusions, global gene expression or chromatin immunoprecipitation) analyses, we found that ^1^O_2_ activated ~160 genes (Fig. 5), with the majority of them the indirect result of this ROS activating a σ factor cascade that includes RpoH_II_, one of two *Rb. sphaeroides* σ^32^ homologs (Green and Donohue, 2006; Dufour *et al.*, 2008; 2012; Ziegelhoffer and Donohue, 2009). Indeed, a combination of *in vitro* and *in vivo* studies showed that < 10% of the ^1^O_2_ activated genes (~13/160 genes) were directly transcribed by σ^E^‐containing RNA polymerase (Anthony *et al.*, 2005; Green and Donohue, 2006; Dufour *et al.*, 2008; 2012; Dufour and Donohue, 2012). Others predict that a larger number of genes are part of the σ^E^‐dependent response to ^1^O_2_ (Glaeser *et al.*, 2007; Nuss *et al.*, 2009; Berghoff *et al.*, 2009), but many studies often do not distinguish direct and indirect effects of ^1^O_2_ on downstream gene expression. The direct targets of *Rb. sphaeroides* σ^E^ encode proteins that could prevent or remove damage from lipid peroxidation, enzymes that can repair mutations, electron transport metalloproteins, another alternative sigma factor RpoH_II_ and proteins of unknown function (Anthony *et al.*, 2005; Green and Donohue, 2006; Watabe *et al.*, 2007; Dufour *et al.*, 2008; 2012; Ziegelhoffer and Donohue, 2009). The finding that *rpoH_II_* transcription is absolutely dependent on σ^E^ (Fig. 5) predicted that ^1^O_2_ activated a transcriptional cascade and that both σ^E^ and RpoH_II_ have a role in this stress response (Anthony *et al.*, 2005; Green and Donohue, 2006; Watabe *et al.*, 2007; Dufour *et al.*, 2008; 2012). As predicted, ^1^O_2_ is bacteriocidal to cells lacking σ^E^ or RpoH_II_ (Anthony *et al.*, 2005; Green and Donohue, 2006; Nuss *et al.*, 2010). Additional direct σ^E^ target genes are needed for rapid ChrR proteolysis, while others encode proteins that could potentially reduce products of olefin oxidation, prevent oxidation of unsaturated fatty acids, serve as electron carriers or repair damaged macromolecules (Dufour *et al.*, 2008; Ziegelhoffer and Donohue, 2009; Lemke *et al.*, 2014).

The ~145 direct RpoH_II_ target genes (Fig. 5) encode bioenergetic enzymes that contain oxidant‐sensitive metal centers (NADH dehydrogenase, etc.), metalloenzymes that synthesize cofactors for bioenergetic enzymes (tetrapyrroles, quinone, etc.) and glutathione‐dependent enzymes that can repair oxidized macromolecules (Dufour *et al.*, 2012; Dufour and Donohue, 2012). Numerous σ^E^ and RpoH_II_ targets have no known function (Dufour *et al.*, 2008; 2012; Dufour and Donohue, 2012), illustrating how little is known about the cellular and biological response to ^1^O_2_ and organoperoxide stress.

Unlike *E. coli*, which contains a single heat shock σ factor (Guisbert *et al.*, 2008), *Rb. sphaeroides* contains two homologs, RpoH_I_ and RpoH_II_ (Karls *et al.*, 1998; Green and Donohue, 2006). ^1^O_2_ is not bacteriocidal to ΔRpoH_I_ cells, and RpoH_I_ activity is increased during heat shock (Karls *et al.*, 1998; Green and Donohue, 2006; Dufour *et al.*, 2012). Indeed, many of the ~130 genes that are directly transcribed by RpoH_I_ encode homologs of typical heat shock proteins (Green and Donohue, 2006; Dufour and Donohue, 2012), so it appears that its primary role is in thermal adaptation, similar to that of *E. coli* σ^32^ (Guisbert *et al.*, 2008). However, many of the 45 genes which are directly transcribed by both RpoH_II_ and RpoH_I_ (Fig. 5) encode proteins that could act in both ^1^O_2_ and heat stress (Green and Donohue, 2006; Dufour *et al.*, 2012; Dufour and Donohue, 2012).

Often, members of a stress regulon are part of a homeostatic loop that is needed to activate the response (Ades *et al.*, 2003; Guisbert *et al.*, 2008). This appears to be true for ^1^O_2_ stress, since mutants lacking σ^E^ target genes that produce furan‐containing fatty acids are defective in increasing activity of this σ factor when they are exposed to this ROS (Nam *et al.*, 2013). However, cells lacking other ECF11 regulon members have normal activation of σ^E^ activity and rates of ChrR proteolysis in the presence of ^1^O_2_ (Hendrischk *et al.*, 2007; Nam *et al.*, 2013; Nuss *et al.*, 2013).

### Conservation of the ECF11 system across the bacterial phylogeny

Selective pressures experienced by cells in nature can dictate a relationship between signals and regulated genes, so the function of a given regulon may have evolved to accommodate variance in environmental conditions across cells with different lifestyles or habitats. For example, a comparative analysis of the *E. coli* σ^E^ regulon in nine γ‐proteobacteria revealed the existence of a ‘core regulon’ that encodes functions involved in envelope stress, plus an ‘extended regulon’ that includes functions related to pathogenesis or symbiosis, and led to the proposal that host‐microbe interactions also activate this stress response (Rhodius *et al.*, 2006).

A similar phylogenetic analysis of σ^E^‐ChrR homologs across bacterial divisions also suggested that this system evolved prior to the divergence of the α‐ and γ‐proteobacteria, and shows that it includes species which have both photosynthetic and non‐photosynthetic lifestyles (Dufour *et al.*, 2008). By analyzing these genomes for genes orthologous to those transcribed by σ^E^ and promoters that contain the motif recognized by this Group IV alternative σ, it was found that many of the direct *Rb. sphaeroides* σ^E^‐ChrR regulon members were present and predicted to contain a σ^E^ promoter in these diverse species. The σ^E^ targets that were most conserved across species, which comprise a so‐called ‘core σ^E^‐ChrR regulon’ of ~8 genes, include the *rpoEchrR* operon and genes involved in synthesis of furan fatty acids that are required for ChrR turnover in the presence of ^1^O_2_ (Dufour *et al.*, 2008). Therefore, it is possible that the photosynthetic and non‐photosynthetic species which contain σ^E^‐ChrR homologs both encounter ^1^O_2_ in nature (Dufour *et al.*, 2008). The observation that proteins required for ChrR turnover in the presence of ^1^O_2_ are conserved members of the core σ^E^ regulon gene suggests that a similar homeostatic feedback loop activates this stress response in other bacteria (Dufour *et al.*, 2008; Nam *et al.*, 2013; Lemke *et al.*, 2014). As predicted by these phylogenetic analyses, the *Caulobacter crescentus* σ^E^‐ChrR system was rapidly activated by ^1^O_2_ and organic hydroperoxides and exhibited a slower response to other inducers (Lourenco and Gomes, 2009), suggesting other signals or pathways activate the ECF11 regulon in this and other species.

Comparative genomics also identified another group of genes directly transcribed by σ^E^ that are not highly conserved among bacterial species and constitute an ‘extended σ^E^‐ChrR regulon’ (Dufour *et al.*, 2008). This extended σ^E^‐ChrR regulon contains numerous genes of unknown function, illustrating the potential to reveal new biology by elucidating their function. There are also gene sets which are only part of the extended σ^E^‐ChrR regulon in selected bacteria, suggesting they encode functions associated with the lifestyle or ecological niche of these organisms (Dufour *et al.*, 2008).

## Future directions

Like other optogenetic circuits (Zhao *et al.*, 2018), one advantage of studying *Rb. sphaeroides* σ^E^‐ChrR is the ease of controlling production of the stimulating signal, ^1^O_2_, by the presence or absence of light (Anthony *et al.*, 2005). In addition, biochemical, genetic, genomic and computational methods were combined to reveal control principles of this system, define processes that are impacted by ^1^O_2_ formation, and predicted the properties of σ^E^‐ChrR networks in other bacteria that contain ECF11 proteins (Newman *et al.*, 1999; Anthony *et al.*, 2003; Campbell *et al.*, 2007; Dufour *et al.*, 2008; 2012; Greenwell *et al.*, 2011; Dufour and Donohue, 2012; Nam *et al.*, 2013; Lemke *et al.*, 2014).

Despite the knowledge accumulated by studying *Rb. sphaeroides* σ^E^‐ChrR, major gaps remain in our understanding on important aspects of its function. For example, to understand how *Rb. sphaeroides* σ^E^ activity is increased, information is needed on the events and proteins that regulate ChrR turnover in the presence of ^1^O_2_. Other needs include insight into a direct interaction of ^1^O_2_, peroxidation products of fatty acids, or other biomolecules with the ChrR‐ASD and ChrR‐CLD, the protease(s) that degrade ChrR, and the signal transduction pathway used to promote turnover of a cytoplasmic anti‐σ factor by a membrane ROS. In addition, identifying the function of genes that are directly transcribed by σ^E^‐containing RNA polymerase but only found in selected species (extended members of the σ^E^ regulon) can provide needed insight into stress response functions associated with lifestyles or ecological niches of these bacteria.

It is crucial to point out that many of the above questions illustrate knowledge gaps for other Group IV alternative σs, so it is likely that answers obtained by analyzing ECF11 proteins will have broad applicability to other regulatory networks. In this way, analysis of the Group IV alternative σs will continue to illuminate new features of biological processes across the bacterial phylogeny.

## Accession numbers

The atomic coordinates for proteins discussed are deposited in the Protein Data Bank (http://wwpdb.org/) under ID codes 2Q1Z, 2Z2S, and 1OR7. The gene expression and chromatin immunoprecipitation data sets discussed can be found in the Gene Expression Omnibus through series accession number GSE39806 (https://www.ncbi.nlm.nih.gov/geo/).
